# Thoracic radiographic features of fatal paraquat intoxication in eleven dogs

**DOI:** 10.1080/01652176.2021.1950945

**Published:** 2021-07-22

**Authors:** Yan-Wun Kuo, Lee-Shuan Lin, Yi-Chia Li, Kuan-Sheng Chen

**Affiliations:** a Department of Veterinary Medicine, College of Veterinary Medicine, National Chung Hsing University, Taichung, Taiwan; b Department of Veterinary Medicine, College of Veterinary Medicine, National Pingtung University of Science and Technology, Pingtung, Taiwan; c Animal Disease Diagnostic Center, National Pingtung University of Science and Technology, Pingtung, Taiwan; d Veterinary Medical Teaching Hospital, College of Veterinary Medicine, National Chung Hsing University, Taichung, Taiwan

**Keywords:** dog, canine, paraquat, radiology, pneumothorax

## Abstract

**Background:**

Paraquat (1,1-dimethyl-4,4-bipyridinium dichloride) is a toxic herbicide. Accidental ingestion of paraquat in animals and humans causes respiratory failure and death.

**Aim:**

To describe the radiographic features of confirmed paraquat intoxication in a group of dogs and determines whether any identified features can facilitate this diagnosis.

**Methods:**

Eleven dogs diagnosed with paraquat intoxication were selected from two institutions between November 2014 and August 2019 comprising five males (all intact) and six females (one intact and five spayed). The mean age was 3.9 ± 2.9 (SD) years and their mean weight was 11.6 ± 5.0 kg. The tentative diagnosis was confirmed through analysis of their urine samples using a colorimetric assay (paraquat concentation 0.39 μg/ml ranging from 0.19-0.65 μg/ml), and their clinical signs were reviewed. Thoracic radiographs were evaluated for the presence of pneumomediastinum, lung patterns (interstitial or alveolar) and their locations (caudodorsal, cranioventral, diffuse, or symmetrical), subcutaneous emphysema, pneumoretroperitoneum, and pneumothorax.

**Results:**

The most common clinical signs were dyspnea (11/11, 100%) and anorexia (9/11, 82%). Pneumomediastinum (10/11, 91%) and symmetrically increased lung opacity (7/11, 65%) were the most common radiographic features. Pneumothorax (3/11, 27%), pleural effusion (3/11, 27%), subcutaneous emphysema (2/11, 18%), and pneumoretroperitoneum (1/5, 20%) were the less common findings. None of the dogs survived.

**Conclusion:**

Pneumomediastinum and diffuse or symmetrical interstitial or alveolar lung patterns are the most common radiographic features in dogs with paraquat intoxication.

**Clinical relevance:**

In countries where this herbicide is not banned, paraquat intoxication should be considered if dogs with no history of trauma present with pneumomediastinum.

## 1. Introduction

Paraquat (1,1-dimethyl-4,4-bipyridinium dichloride) is a toxic herbicide first introduced in 1962 and used globally (Vale et al. 1987). Its unique properties, such as its fast and strong effect upon contact with leaves and its immediate inactivation on soil, made it one of the most popular herbicides in some countries (Conning et al. 1969). In contrast to the scenario decades ago, most recent cases of paraquat poisoning in humans occur due to attempted suicide rather than accidental ingestion (Vale et al. 1987). Paraquat can induce poisoning in the gastrointestinal tract, damage the kidney and the heart, and accumulate in the lungs (Smith and Heath 1976).. In England and other European countries, paraquat has been withdrawn from the market; however, it is still available in some countries in Asia and Africa (Kervégant et al. 2013; Williams et al. 2016).

Paraquat intoxication in humans is mostly caused by accidental ingestion or deliberate self-poisoning of the liquid paraquat formulation (Dinis-Oliveira et al. 2008). Inhalation and skin contact can also serve as routes of poisoning. In contrast, besides accidental ingestion, paraquat poisoning in dogs and cats can also result from deliberate poisoning of baited food (Longstaffe et al. 1981). In both humans and animals, the end result of paraquat poisoning is usually death consequent to severely impaired respiratory function (Longstaffe et al. 1981; Dinis-Oliveira et al. 2008).

In studies of various species of experimental animals such as rats, mice, dogs, and monkeys, the pulmonary lesions were similar to those reported in humans (Dinis-Oliveira et al. 2008). The lung shows time-dependent accumulation of paraquat in the alveolar epithelial cells and the Clara cells (Smith and Nemery 1987). The development of pulmonary lesions is classified into two phases (Smith et al. 1974; Smith and Heath 1976). The initial phase is the “destructive phase.” One to three days after intoxication, type I and type II alveolar epithelial cells show swelling, vacuolation, and disruption of mitochondria and endoplasmic reticula (Kimbrough and Gaines 1970; Sykes et al. 1977). Afterwards, cytoplasm expanding into the alveolar space and the rupture of type I pneumocytes lead to the exposure of the basement membrane (Smith and Heath 1976). The second phase is the “proliferative phase” (Dinis-Oliveira et al. 2008). The primary finding in this stage is extensive fibrosis, considered a reparative reaction to the damaged pneumocytes. Fibrosis destroys the normal lung structures and leads to hypoxemia (Fukuda et al. 1985). On necropsy, the lungs typically appear heavy, dark, and rubbery with signs of hemorrhage and consolidation; pneumomediastinum has also been reported (Kelly et al. 1978). Microscopically, alveolar capillary congestion, edema, and collapse of alveolar ducts and terminal bronchioles are initially observed. Afterwards, alveolar fibrosis and bronchiolar epithelial hyperplasia are evident (Kelly et al. 1978).

It can be challenging to evaluate the respiratory distress of dogs and cats in emergencies (Thrall 2018; Ward et al. 2018). Thoracic radiography is a non-invasive and readily available diagnostic modality of considerable clinical value. Although it only provides two-dimensional images, it remains the first option when imaging of the thorax is required (Prather et al. 2005; Thrall 2018). In a study of 42 human patients with paraquat poisoning, their thoracic radiographs showed consolidation, pneumomediastinum, pneumothorax, subcutaneous emphysema, cardiomegaly, and pleural effusion; the mortality rate of the patients who had pneumomediastinum was nearly 100% (Im et al. 1991). The veterinary radiologic description of paraquat intoxication is limited; this study aimed to describe the radiographic features of confirmed intoxication in dogs. We hypothesized that identifying specific radiographic findings may facilitate the diagnosis of paraquat intoxication in dogs.

## 2. Materials and methods

This was a retrospective study. From 2014 to 2019, eleven dogs diagnosed with paraquat intoxication were selected from two veterinary medical teaching hospitals in Taiwan, the National Chung Hsing University and the National Pingtung University of Science and Technology. The history and the medical record of each patient was reviewed. Information obtained from the medical records included breed, sex, age, body weight, history, clinical signs, clinicopathological data, and outcome. The inclusion criteria were as follows: (1) diagnosis of paraquat poisoning was confirmed by urine analysis, (2) the concentration of paraquat in the urine sample was recorded, and (3) thoracic radiographs of at least two orthogonal projections at the time of admission.

All the urine samples were collected via ultrasound-guided (SSA-660A, Toshiba, Japan) cystocentesis. The urine samples were stored at temperatures between 4 and 8 °C, and sent to the Department of Clinical Toxicology and Occupational Medicine, Taipei Veterans General Hospital, Taiwan, for analysis of the paraquat concentration using a colorimetric assay (Scherrmann et al. 1987).

All images were taken at the end of inspiration using digital radiography (MRAD-A50S Xray generator, Toshiba, Japan; CXDI-70C flat panel detector, Canon, Japan), and were reviewed using a medical image viewer software (SoliPACS^TM^ Web Viewer, EBM Technologies, Taipei, Taiwan). The radiographs were reviewed and assessed by consensus of two observers, a radiologist (KSC) with 16 years of experience, and a radiology graduate student (YWK) with 2 years of experience in radiology. Images were specially evaluated for the presence of pneumomediastinum, type and location of lung pattern, subcutaneous emphysema, pneumoretroperitoneum, and pneumothorax. Lung pattern was characterized as interstitial, alveolar, or bronchial. The severity of the radiographic features of pneumomediastinum, pneumothorax, subcutaneous emphysema, retroperitoneal emphysema, pleural effusion, interstitial and alveolar pattern was classified as mild or severe. Gas in the pleural or subcutaneous space was defined as “mild” if gas pockets were visible or “severe” when a large amount of gas was present. Gas in the mediastinal or retroperitoneal space was defined as “mild” when linear radiolucencies delineated the adventitial surface of the trachea or abdominal aorta and “severe” when large mediastinal vessels or kidneys were outlined by gas. Fluid in the pleural space was defined as “mild” if thickened interlobar fissures were visible or “severe” when retraction of lungs was present from the thoracic wall. “Mild” interstitial pattern was defined as increased pulmonary intensity with relatively distinct pulmonary vessels compared with “severe” interstitial pattern with indistinct pulmonary vessels. “Mild” alveolar pattern was defined as partial pulmonary vessels obscured by focal increased lung opacities, while “severe” alveolar pattern was defined as increased lung opacity completely obscuring pulmonary vessels. The bronchial pattern was defined by whether thickened bronchial walls were present. The distribution of lung patterns was categorized as diffuse, symmetrical, or asymmetrical in caudodorsal or cranioventral lungs.

The data were analyzed using the commercially available software, SAS (Version 9.4, SAS institute Inc, Cary, NC, USA). The Shapiro-Wilk normality test was used to determine whether continuous variables were normal distributions. For continuous variables (age, body weight, and concentration of paraquat in the urine samples), descriptive statistics such as mean ± standard deviation (variables normally distributed) or median with interquartile range (variables not normally distributed) were calculated. Moreover, categorical variables (breed, sex, clinicopathological data, presence of pneumomediastinum, type and location of lung pattern, subcutaneous emphysema, pneumoretroperitoneum, and pneumothorax) were presented in a tabular form. Percentages were computed for categorical data.

## 3. Results

Eleven dogs met the inclusion criteria of this study. Five of the dogs were male (all intact) and six were female (one intact and five spayed). Accidental or intentional paraquat intoxication in these dogs could not be determined because they were outdoor dogs and lived in the rural area. Variables of age and body weight were normally distributed (p > 0.05), but variables of urine paraquat concentration were not normally distributed. The mean age was 3.9 ± 2.9 years and their mean weight was 11.6 ± 5.0 kg. Eight dogs were mixed breeds (8/11, 73%), whereas three were purebred dogs, including a Shiba (1/11, 9%), a Dachshund (1/11, 9%), and a Miniature poodle (1/11, 9%). The clinical signs included dyspnea (11/11, 100%), anorexia (9/11, 82%), vomiting (4/11, 36%), trembling (1/11, 9%), brick-red mucous membrane (1/11, 9%) (Ware 2014), cyanosis (1/11, 9%), dehydration 5-8% (3/11, 27%), and capillary refill time > 2 seconds (1/11, 9%). No signs of diarrhea were observed in all dogs.

Complete blood count (CBC) was performed on ten dogs and biochemical profile tests were performed on eight dogs upon admission. Biochemistry and CBC were not performed on one dog, and biochemistry was not performed on two dogs because the owners refused to allow further examinations to be performed when they were told the tentative diagnosis of paraquat intoxication in their dogs. Complete blood count abnormalities revealed thrombocytopenia (5/10, 50%), leukocytosis (3/10, 30%), mild to moderate anemia (1/10, 10%) and thrombocytosis (1/10, 10%). All eight dogs that underwent biochemical profile tests had hypochloremia (8/8, 100%); other biochemical abnormalities observed included hyperglycemia (7/8, 88%), hypercalcemia (5/6, 83%), elevated blood urea nitrogen (6/8, 75%) and creatinine (3/8, 38%), hyperproteinemia (5/8, 63%), hyperglobulinemia (5/8, 63%), hypokalemia (5/8, 63%), hyperphosphatemia (3/6, 50%), hypomagnesemia (3/6, 50%), hyponatremia (3/8, 38%), hyperalbuminemia (2/8, 25%), elevated alkaline phosphatase activity (2/8, 25%), hyperbilirubinemia (1/6, 17%), decreased activity of alanine transferase (1/8, 13%), and elevated activity of alanine transferase (1/8, 13%).

Urine samples were collected from all 11 dogs. In eight dogs, the median concentration of the paraquat detected in the samples was 0.39 μg/ml (range: 0.19-0.65 μg/ml). In three dogs, the level of paraquat was too low to be accurately quantified (< 0.1 μg/ml).

**Figure 1.**
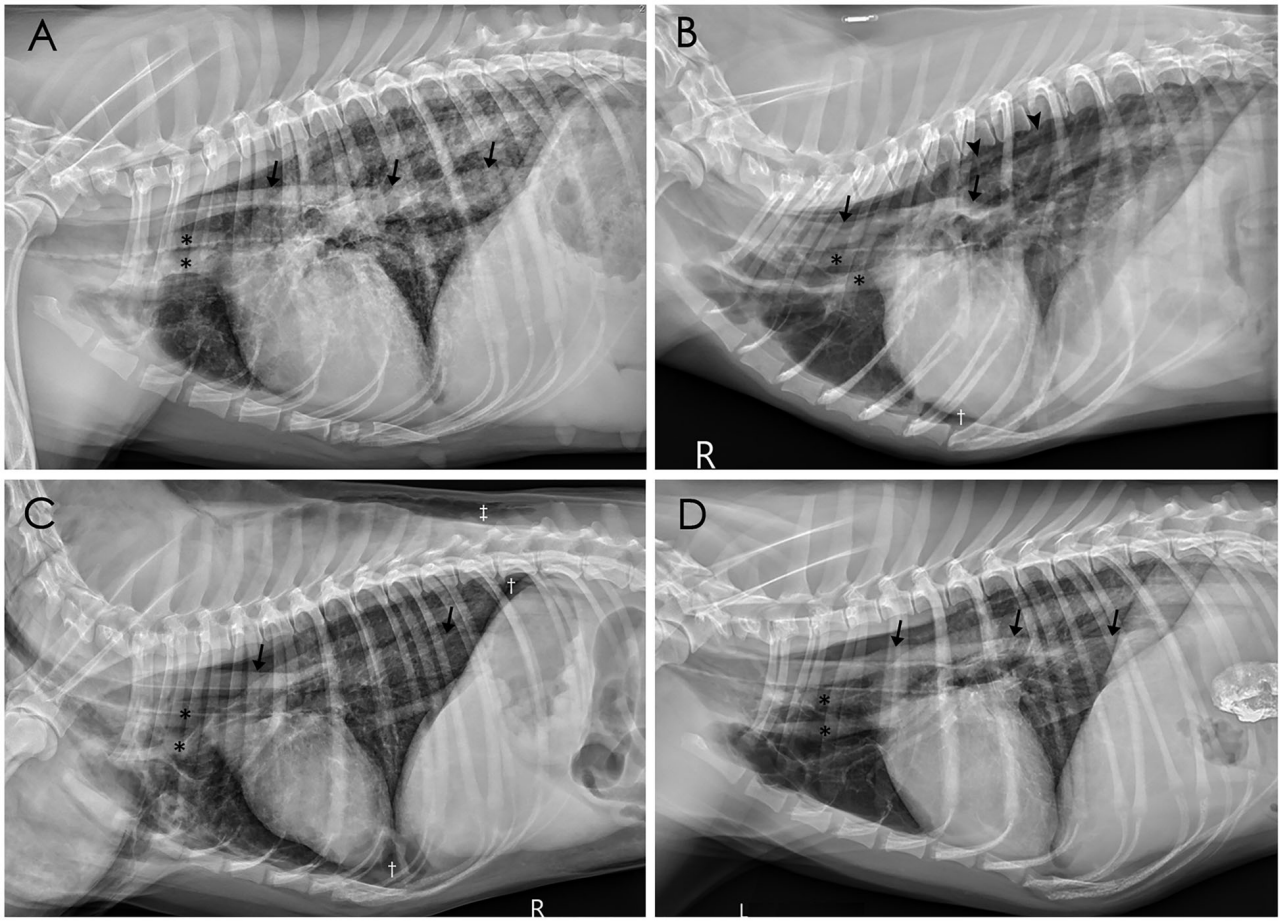
Right (A, B, C) and left (D) lateral thoracic radiographs of Dogs 3, 4, 6, 7, respectively, showing pneumomediastinum. The adventitial surface of the trachea, esophagus (arrowheads), and cranial mediastinal vessels (✽) are visible due to the presence of gas in the mediastinum (A-D). The azygos veins (arrow heads) are also visible (B). Mild pneumothorax († in B, C) and subcutaneous emphysema (‡ in C) were observed.

**Figure 2.**
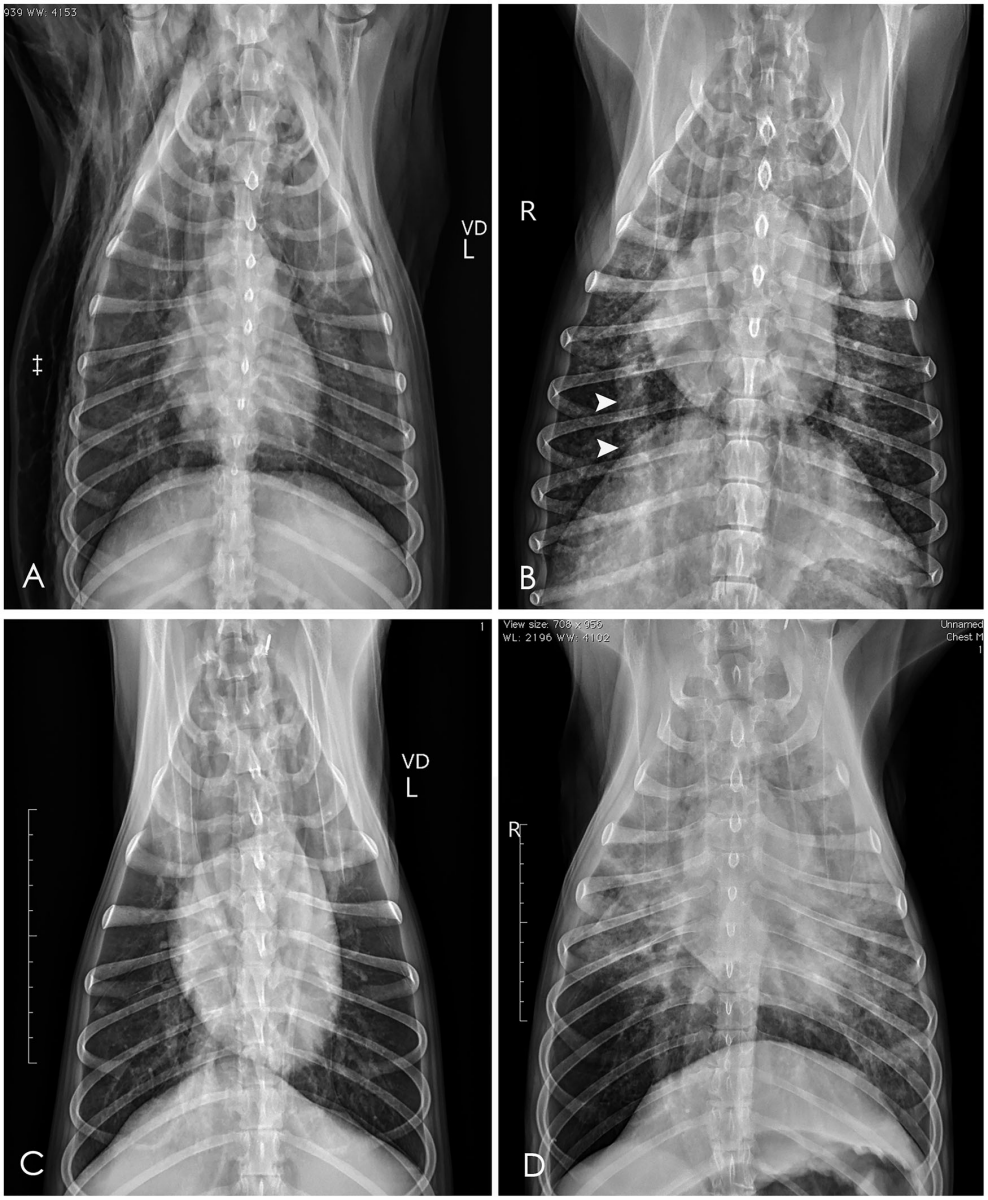
Illustration of symmetrical diffuse interstitial lung pattern seen on the radiographs of the ventrodorsal (A, Dog 6) and dorsoventral (B, dog 3) lungs with enlargement of right ventricle and pulmonary arteries (arrow heads). Severe subcutaneous emphysema was also observed in Dog 6 (‡). Mildly increased interstitial pattern was observed in both caudodorsal lungs of Dog 7 (C). Illustration of the asymmetrical distribution of interstitial and alveolar lung patterns in the radiographs of the ventrodorsal lungs of Dog 1 (D).

The radiographic findings are summarized in Table 1. The days between the onset of clinical signs to radiography ranged from 1 to 7 days. Pneumomediastinum (Figure 1) was the most common radiographic finding (10/11, 91%), and was detected in two dogs after two days of hospitalization. Symmetrically increased opacity of lung was another common radiographic finding (7/11, 64%); the rest of the patients had asymmetrical distributions (Figure 2.). The caudodorsal lungs were most commonly affected with symmetrical distributions (8/11, 73%, Table 2). Other radiographic findings included bronchial patterns (3/11, 27%), pneumothorax (3/11, 27%), pleural effusion (3/11, 27%), and subcutaneous emphysema (2/11, 18%). Abdominal radiography was performed on only five dogs, and pneumoretroperitoneum was observed in one dog.

**Table 1.** Radiographic features of paraquat intoxication in 11 dogs.

**Dog** **no.**	Age	Sex	Breed	Interstitial pattern	Alveolar pattern	Bronchial pattern	PM	PT	SE	PRP	PE	Days^†^
**1**	7	FS	Mix	Lt caudodoral, severe	Cranioventral, symmetrical, severe	N	N	N	N	N	Mild	2
**2**	4	M	Mix	Caudodorsal, symmetrical, mild/ N *	N/Severe*†	N/N*	N/ Severe*	N/N*	N/N*	N/A	N/N*	2
**3**	10	FS	Mix	Diffuse, severe	N	V	Severe	N	N	N/A	N	1
**4**	4	F	Dachshund	Cranioventral, symmetrical, mild	Right caudodorsal, severe	V	Severe	Mild	N	Mild	N	3
**5**	2	M	Mix	Caudodorsal, symmetrical, mild; Rt cranioventral, severe	Left cranioventral, severe	N	Severe	N	Mild	N/A	N	5
**6**	1	FS	Mix	Diffuse, mild	N	N	Severe	Mild	Severe	N	Mild	6
**7**	2	M	Shiba	Caudodorsal, symmetrical, mild	N	N	Severe	N	N	N	N	7
**8**	3	FS	Mix	Caudodorsal, symmetrical, mild	N	N	Mild	N	N	N/A	N	4
**9**	1	FS	Mix	Lt cranioventral, mild/ Lt cranioventral, severe *	Rt cranioventral, mild/ Rt cranioventral, severe*	N/N*	N/ Severe*	N/ Mild*	N/N*	N/N*	N/N*	2
**10**	7	M	Miniature poodle	Caudodorsal, symmetrical, mild	Cranioventral, symmetrical, severe	N	Severe	N	N	N/A	Mild	3
**11**	2	M	Mix	Diffuse, severe	N	V	Severe	N	N	N/A	N	5

*, 2 days later. †, only one lateral radiograph was taken. F, female. FS, female spayed. M, male. V, visible. N, not visible. N/A, not applicable (abdominal radiography did not perform). Lt, left. Rt, right. PM, pneumomediastinum. PT, pneumothorax. SE, subcutaneous emphysema. PRP, pneumoretroperitoneum. PE, pleural effusion

^†^, days from the onset of clinical signs to radiography

**Table 2.** Distribution of lung pattern.

	Interstitial pattern		Alveolar pattern	
Lungs*	Symmetrical	Asymmetrical	Symmetrical	Asymmetrical
Caudodorsal	5	1	0	1
Cranioventral	1	2	2	2
Diffuse	3	–	0	–

*Evaluation of radiography on the first day of presentation.

**Figure 3.**
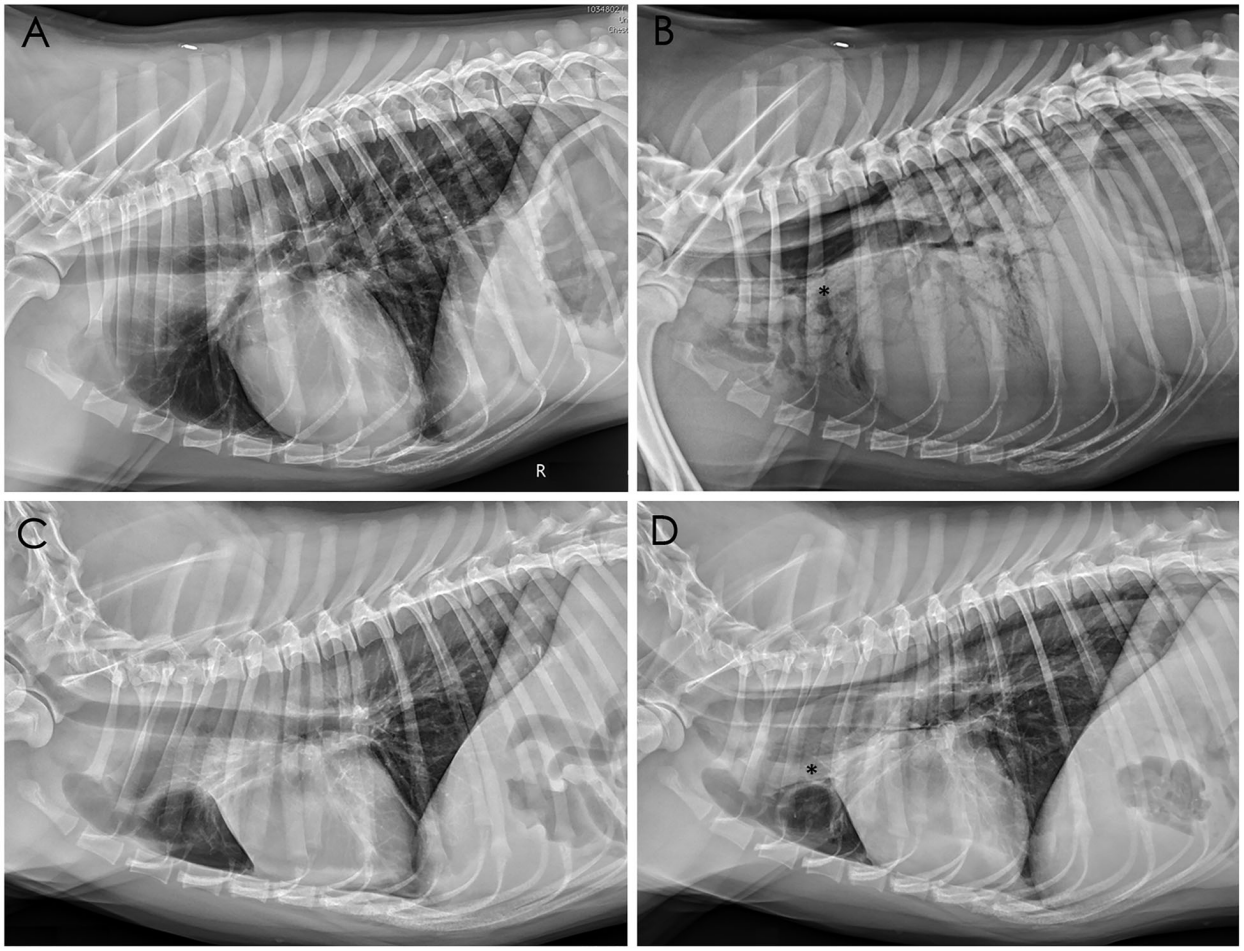
Radiographs of the right (A, B) and left (C, D) lateral projections of Dogs 2 and 9, respectively. No pneumomediastinum was observed on the day of presentation (A, C). Radiography performed two days later showed pneumomediastinum with visible adventitial margins of the trachea and cranial mediastinal vessels (✽in B, D). Severe alveolar pattern was also observed in the whole lungs of Dog 2 (B).

Pneumomediastinum was detected in Dogs 2 and 9 after two days of hospitalization. On the first day of presentation, Dog 2 showed dyspnea and anorexia. Its thoracic radiography images showed a mild unstructured interstitial lung pattern in the caudodorsal lungs. However, marked pneumomediastinum and severe alveolar lung pattern were observed throughout the lungs two days later (Figure 3A and B). A similar scenario was observed on Dog 9, who also presented with a history of dyspnea, vomiting, and trembling muscles. Pneumomediastinum was not observed on its thoracic radiographs until two days later (Figure 3C and D).

There were no survivors in this study. Five dogs were euthanized due to rapid deterioration and the rest died during supportive treatment.

## 4. Discussion

This study showed that pneumomediastinum is the most common radiographic feature of paraquat intoxication in dogs. However, this finding is rarely mentioned in veterinary studies (Cope et al. 2004; Dinis-Oliveira et al. 2008). Pneumomediastinum is defined as presence of free air or gas within the mediastinum due to various intrathoracic or extrathoracic causes, such as trauma, rupture of a hollow organ, intrathoracic infections by gas-forming organisms or interventions in the esophagus or tracheobronchial tree (Newcomb and Clarke 2005). The gas in the mediastinal space provides negative contrast for the radiographic visualization of the mediastinal structures. The pathogenesis of pneumomediastinum was first demonstrated in 1937 (Macklin 1937). It was believed that alveolar rupture beneath an intact visceral pleura could release air into the mediastinum through the peribronchial and perivascular adventitia (so called Macklin effect) (Macklin 1937). The air, which then migrates into the neck, skin, or retroperitoneum through diaphragmatic aortic hiatus, may cause subcutaneous emphysema and pneumoretroperitoneum. Increased mediastinal pressure by free air or gas can also lead to rupture of the visceral pleura; if the air extends into the pleural space, pneumothorax subsequently occurs (Cochran et al. 1994; Agut et al. 2015).

In this study, paraquat intoxication is also characterized by symmetrically increased and unstructured interstitial opacity in the caudodorsal lungs. Differential diagnoses for unstructured interstitial opacity include interstitial pneumonia, interstitial inflammation, pulmonary edema, hemorrhage, and fibrosis (Dennis et al. 2010). Although echocardiography was not performed in this study, no left heart enlargement was observed radiographically in any of the dogs; therefore, the unstructured interstitial opacity is more likely associated with non-cardiogenic pulmonary edema than a cardiogenic one. Increased interstitial and alveolar opacities in the cranioventral lungs were also observed in five dogs. Since vomiting is a common clinical sign of paraquat poisoning in dogs, concurrent aspiration pneumonia in the cranioventral lungs should be considered as another differential diagnosis.

Pulmonary emphysema was confirmed in Dog 11 histopathologically. It is defined as the permanent dilatation of the distal airways as a result of the destruction of alveolar architecture without fibrotic changes (Devasahayam et al. 2020). In paraquat poisoning, pulmonary emphysema with largely distended alveolar spaces and terminal bronchioles and loss of the integrity of the alveolar walls can be observed histopathologically (Manktelow 1967; Matthew et al. 1968). Pulmonary emphysema has been reported as a probable cause of pneumomediastinum in a dog with confirmed congenital pulmonary emphysema (Stephens et al. 2002). The pressure difference occurs as of result of rupturing of alveoli between the mediastinum and the peripheral lung parenchyma, which leads to the air dissecting along the peribronchial connective tissues and subsequently into the mediastinum (Stephens et al. 2002). It was difficult to detect pulmonary emphysema radiographically in this study since radiography is not as sensitive as computed tomography in the detection of pulmonary emphysema (Washko 2010; Gil et al. 2014; Devasahayam et al. 2020). To detect pulmonary emphysema, radiography should be performed at full inspiration and expiration to observe whether there is no difference in the lung opacity (Dennis et al. 2010). However, this technique was not performed on these dyspneic dogs in this study because it was difficult to take the radiographs at the precise time of full inspiration and expiration.

Heartworm infestation was confirmed in Dog 3 by the commercialized heartworm antigen test kit (SNAP Heartworm RT test, IDEXX, Westbrook, ME, USA), and enlargement of the right ventricle and pulmonary arteries were detected radiographically (Polizopoulou et al. 2000). The dog had a diffuse unstructured interstitial lung pattern, which could be attributed to interstitial inflammatory response due to heartworm infestation (Carlisle 1980). However, the thoracic radiographs of this dog also showed pneumomediastinum, suggesting that paraquat intoxication could be another differential diagnosis for diffuse unstructured interstitial lung pattern and should be considered in such cases.

Ingestion of paraquat usually leads to gastrointestinal toxicity. Evidence of gastrointestinal irritation, necrosis, ulceration of pharyngeal epithelium, and edema of the stomach wall has been observed in cases of paraquat poisoning (Kelly et al. 1978). In a case series of seven dogs with paraquat poisoning, one of the dogs that presented with vomiting and gas-filled bowel loops was presumptively diagnosed with gastroenteritis (Cope et al. 2004). Therefore, combined with clinical signs such as vomiting and anorexia, paraquat intoxication may be misdiagnosed as gastrointestinal disease. This could account for the challenge to make an early diagnosis of paraquat intoxication. In the present study, both anorexia and vomiting were common clinical signs. Although all the owners did not observe how the dogs became intoxicated, ingestion was the most likely route. Two dogs (Dogs 5 and 6), with a history of anorexia and vomiting for 3 and 5 days, respectively, were initially suspected as having foreign bodies or gastroenteritis at other veterinary clinics before they were referred to the teaching hospital. In the countries where the use of paraquat is not banned, paraquat intoxication should be considered if dogs present with vomiting, anorexia, and respiratory distress.

Aggressive early decontamination to decrease absorption is key for successful treatment of an acute paraquat poisoning (Dinis-Oliveira et al. 2008). Oral administration of absorbents, such as 15% Fuller’s earth together with magnesium sulfate or activated charcoal, should be performed preferably within 60 minutes, but no longer than 4-5 hours, to neutralize the ingested paraquat (Akintonwa et al. 1984). Activated charcoal has been reported to be effective on paraquat absorption (Gaudreault et al. 1985). No absorbents were administered in the patients in this study because the timing for early treatment to neutralize the poison had passed when the patients were sent to the hospitals.

Anti-inflammatory and immunosuppressant drugs have been known to reduce pulmonary inflammation and fibrosis caused by paraquat intoxication (Eddleston et al. 2003). It has been found that leukopenia induced by cyclophosphamide may reduce the severity of lung inflammatory process of paraquat poisoning (Addo and Poon-King 1986). Anti-inflammatory treatment with repeated pulse therapy of cyclophosphamide and methylprednisolone was reported to reduce the mortality rate in humans with severe paraquat poisoning (Lin et al. 2006). More recently, intravenous cyclophosphamide and methylprednisolone were reported to be potential agents for improving patient survival (Koh et al. 2014). Moreover, a methylprednisolone and dexamethasone combination has been used successfully in human clinical cases of paraquat intoxication (Chen et al. 2002). Prednisolone was administered to four dogs, and cyclophosphamide along with prednisolone was administered to one dog in this study. However, these treatments appeared ineffective probably because of the late stage of intoxication and rapidly deteriorating condition of the patient upon arrival to the hospital.

The primary limitation of the present study is its retrospective nature. In addition, only one dog underwent necropsy and histopathological examination. Thus, the histopathological results may not completely reflect all the radiographic findings. Another limitation is the small size of the study population. Nevertheless, the findings of this study provide useful radiographic information that can facilitate the diagnosis of paraquat intoxication if dogs present sudden dyspnea and pneumomediastinum.

In conclusion, if pneumomediastinum in combination with increased interstitial and alveolar lung opacities are detected on the thoracic radiographs of dogs without a history of trauma, further urine analysis is warranted to confirm paraquat intoxication, especially in countries that allow the use of paraquat.
